# Wisteria floribunda agglutinin-positive Mac-2-binding protein in the prediction of disease severity in chronic hepatitis B patients

**DOI:** 10.1371/journal.pone.0220663

**Published:** 2019-08-08

**Authors:** Ming-Lun Yeh, Chung-Feng Huang, Ching-I Huang, Chia-Yen Dai, I-Hung Lin, Po-Cheng Liang, Meng-Hsuan Hsieh, Zu-Yau Lin, Shinn-Chern Chen, Jee-Fu Huang, Jyh-Jou Chen, Ming-Lung Yu, Wan-Long Chuang

**Affiliations:** 1 Hepatitis Center and Hepatobiliary Division, Department of Internal Medicine, Kaohsiung Medical University Hospital, Kaohsiung, Taiwan; 2 Faculty of Internal Medicine, College of Medicine, Kaohsiung Medical University, Kaohsiung, Taiwan; 3 Health Management Center, Kaohsiung Medical University Hospital, Kaohsiung, Taiwan; 4 Division of Gastroenterology and Hepatology, Chi Mei Medical Center, Liouying, Taiwan; Nihon University School of Medicine, JAPAN

## Abstract

**Background:**

Wisteria floribunda agglutinin-positive Mac-2-binding protein (WFA^+^-M2BP) was a novel marker of liver fibrosis. We aimed to investigate WFA^+^-M2BP level in assessing liver fibrosis in patients with chronic hepatitis B (CHB) infection.

**Methods:**

A total of 160 CHB patients, who received a liver biopsy, were consecutively recruited. Serum WFA^+^-M2BP level was quantified at the time point of biopsy. The results were compared with histopathological manifestations and clinical characteristics of the patients.

**Results:**

The median WFA^+^-M2BP level, aspartate aminotransferase-to-platelet ratio (APRI) and Fibrosis-4 (FIB-4) index were 1.20 COI, 1.19, and 1.63, respectively. Fifty-one (31.9%) patients had advanced fibrosis. There was a significant increase of WFA^+^-M2BP levels in parallel to necroinflammation/fibrosis stages. The areas under the receiver operating characteristic curve (AUROC) of WFA^+^-M2BP level for predicting fibrosis stages were 0.780 of F2, 0.785 of F3, and 0.769 of F4, respectively (all p <0.001). The multivariate analysis identified age (Odds ratio [OR] 1.05, 95% confidence interval [CI]: 1.010–1.092, p = 0.014), platelet (OR: 0.99, 95%CI: 0.980–0.998, p = 0.013), and WFA^+^-M2BP level (OR: 1.97, 95% CI: 1.299–2.984, p = 0.001) as independent factors associated with advanced fibrosis. Combination of age, platelet and WFA^+^-M2BP level achieved a better diagnostic performance for advanced fibrosis (AUROC: 0.732, accuracy: 81.3%) than APRI (AUROC: 0.577, accuracy: 63.8%) or FIB-4 index (AUROC: 0.691, accuracy: 75.6%).

**Conclusion:**

WFA^+^-M2BP had a good performance indistinguishing liver fibrosis in CHB patients. The combination of age, platelet, and WFA^+^-M2BPaddressed more accuracy in identifying patients with advanced fibrosis.

## Introduction

Hepatitis B virus (HBV) infection remained the leading cause of liver cirrhosis (LC) and hepatocellular carcinoma (HCC) worldwide, especially in the Asia-Pacific region.[1]HBV viral load, hepatitis B e antigen (HBeAg) and liver fibrosis severity were associated with the development of LC and HCC in patients with chronic HBV infection (CHB).[2,3] With long-term HBV antiviral therapy, nucleos(t)ide analogs, the risk of LC and HCC decreased and regression of fibrosis was also achieved.[4,5]The current regional guidelines recommended HBV antiviral therapy in patients with high HBV viral load with significant fibrosis (Metavir≥ F2), even the alanine aminotransferase (ALT) level was below the 2-fold upper limit of normal (ULN).[6,7]Therefore, disease severity assessment, namely fibrosis staging, is essentialin the decision-making of antiviral therapy as well as complication evaluation in a clinical setting.

Fibrosis development is a subtle process during the natural course of CHB. Significant fibrosis development is an important checkpoint during fibrogenesis, and which implies that antiviral therapy should be implemented.[8]Liver biopsy is the current gold standard to assess the severity of necroinflammation and fibrosis.[9] However, liver biopsy was largely limited by the invasiveness and potential complications.[10]In addition, the sampling error, and inter-observer discrepancy were other limitations for the accuracy of liver biopsy. Recently, several non-invasive tools, including mechanical and serological methods, for liver fibrosisassessment have been vigorously developed.[11]Among the serological methods, aspartate aminotransferase (AST)-to-platelet ratio (APRI) index and Fibrosis-4 (FIB-4) index werecommonly used and were easily assessed. However, the predictive value in CHB remained uncertain.[12,13]Wisteria floribunda agglutinin-positive Mac-2-binding protein (WFA^+^-M2BP), a product released during fibrogenesis process, was recently developed as a novel serological marker of liver fibrosis.[14,15]Recent studies demonstrated a significant association between serum WFA^+^-M2BP level and severity of liver fibrosis in subjects with hepatitis C virus infection and non-alcoholic steatohepatitis.[16–18] The comparison of WFA^+^-M2BPand directly fibrosis assessment, Fibroscan, had also been studied. In the recent study investigating a large scale of chronic hepatitis C patients, the authors demonstrated that serum WFA^+^-M2BP was superior to other non-invasive markers in the prediction of significant fibrosis and had the greatest specificity in the diagnosis of cirrhosis.[19]In addition, the serum WFA^+^-M2BP level could predict the development of liver-related events, including the occurrence of decompensation and the development of HCC.[16,20,21]However, application of WFA^+^-M2BP level in assessing liverfibrosis has rarely been investigatedin CHB patients.

Consequently, we conducted the study aimed to investigate the performance of serum WFA^+^-M2BP level in the prediction of liver fibrosis in CHB patients. We also aimed to explore anaccurate predictive model for significant fibrosis in CHB.

## Materials and methods

The study was conducted according to the guidelines of the Declaration of Helsinki and was approved by the ethics committee of Kaohsiung Medical University Hospital (KMUHIRB-E(II)-20150130). Written informed consent was obtained from all of the patients. CHB patients who received a liver biopsy prior to antiviral therapy during 2000 and 2015, were recruited in the present study. The inclusion criteria included 1). Positive serum hepatitis B surface antigen (HBsAg) for more than 6 months; 2).The results of the liver biopsy consistent with the diagnosis of CHB based on the severity of necroinflammation and fibrosis. The exclusion criteria included the followings: hepatitis C virus or human immunodeficiency virus co-infection; evidence of other liver diseases such as α-1 anti-trypsin deficiency, hemochromatosis, Wilson’s disease, etc.

The liver biochemistry, HBV serological markers, and HBV DNA level were testedat enrollment. HBsAg and HBeAg were detected by commercially available enzyme-linked immunosorbent assay kits (Abbott Laboratories, North Chicago, IL, USA). The serum HBV DNA levels were determined using a CobasAmpliPrep/CobasTaqMan HBV assay (CAP/CTM, version 2.0, Roche Diagnostics, Indianapolis, IN, USA; the dynamic range was 20 IU/mL– 1.7x10^8^IU/mL).APRI and FIB-4 index were calculated using the formula: APRI = AST [/ULN] x 100/platelet count (x10^9^/L)[22]; FIB-4 = age (years) x AST [U/L]/(platelet count [10^9^/L] x (ALT [U/L]^1/2^).[23]

### Measurement of WFA^+^-M2BP

Stored serum obtained at the same time of liver biopsy was used for WFA^+^-M2BP measurement. Serum WFA^+^-M2BP level was quantified by lectin-Ab sandwichimmunoassay, using an automatedimmunoanalyzer (HISCL-800; Sysmex Co, Hyogo, Japan).[15] The measured WFA^+^-M2BP levels werefurther indexed using the following equation:

Cutoff index (COI) = ([WFA^+^-M2BP]_sample_- [WFA^+^-M2BP]_NC_)/([WFA^+^-M2BP]_PC_- [WFA^+^-M2BP]_NC_)

Where [WFA^+^-M2BP]_sample_ means WFA^+^-M2BP level of the serum sample, NC means negative control, and PC means positive control.

### Histopathology

All enrolled patients receivedultrasound-guided liver core biopsy using a 16- or 18-gauge biopsy needle. Two experienced hepato-pathologists reviewed and determined the grade of necroinflammation (A0–3) and stage of fibrosis (F0–4) for each specimen according to the METAVIR scoring system.[24] The significantfibrosis was defined as ≥F2 and advanced fibrosis was ≥F3.

### Statistical analysis

Descriptive statistics were computed for all of the variables, including the median and 25^th^, 75^th^ percentile for continuous variables. For the categorical variables, the frequencies and percentages were estimated. The Mann-Whitney U test was used to compare continuous variables and the chi-squared and Fisher’s exact tests were used to compare categorical variables. Binary logistic regression analysis was used to identify the independent factors associated with significant fibrosis. Kruskal-Wallis H test was used to comparing serum WFA^+^-M2BP level between different grades of necroinflammation and stages of fibrosis. The Spearman rank correlation coefficient test was used for the correlation between continuous variables and WFA^+^-M2BP level. Area under the receiver operating characteristics curve (AUROC) analysis wasdone to evaluate the diagnostic accuracy of WFA+-M2BP, APRI and FIB-4 index for liver fibrosis. The optimal cutoff value for fibrosis stage was determined by the Youden’s index. All tests were two-sided, and p <0.05 was considered significant. All analyses were performed using the SPSS 19.0statistical package (SPSS Inc., Chicago, IL, USA).

## Results

### Patients’ characteristics

A total of 160 CHB patients (123 males, median age 40 years) were consecutively recruited. Table 1 showed the demographic characteristics of thepatients. Fifty-six (35.0%)of the patients were HBeAg positive. The mean HBV DNA level was 5.9log_10_IU/ml. The median platelet, AST, and ALT levels were 182.0 x 10^3^/uL, 77.5, and 133.0U/L, respectively. The mean serum WFA^+^-M2BP level, APRI, and FIB-4 index were 1.20, 1.19, and 1.63, respectively. The histopathologicalresults revealed 51.9% and 55.0% of patients were ofsignificant necroinflammation and significant fibrosis, respectively.

**Table 1:** Characteristics of all the 160 patients with chronic HBV infection.

	All (N = 160)
Age, years	40.0 (30.3, 52.0)
Male gender	123 (76.9)
platelet, x10^3^/μl	182.0 (140.3, 225.8)
AST, U/L	77.5 (54.0, 144.5)
ALT, U/L	133.0 (83.0, 243.8)
Positive HBeAg	56 (35.0)
HBV DNA, log_10_ IU/ml	5.9 (4.1, 7.4)
WFA^+^-M2BP, COI	1.20 (0.54, 1.99)
APRI	1.19 (0.67, 2.31)
FIB-4	1.63 (0.90, 3.33)
Necro-inflammation grade	
A0	48 (30.0)
A1	29 (18.1)
A2	54 (33.8)
A3	29 (18.1)
Fibrosis stage	
F0	21 (13.1)
F1	51 (31.9)
F2	37 (23.1)
F3	25 (15.6)
F4	26 (16.3)

AST, aspartate aminotransferase; ALT, alanine aminotransferase; HBeAg, hepatitis B e antigen; WFA^+^-M2BP, wisteria floribunda agglutinin positive Mac-2-binding protein; APRI, aspartate aminotransferase-to-platelet ratio; FIB-4, Fibrosis-4.

### Correlation between WFA^+^-M2BP level and laboratory parameters

The serum WFA^+^-M2BP level was significantly correlated with age (r = 0.354, p <0.001, 95% CI 0.033–0.080), AST (r = 0.179, p = 0.023, 95% CI 0.000–0.003), APRI (r = 0.273, p <0.001, 95% CI 0.052–0.180), FIB-4 index (r = 0.484, p <0.001, 95% CI 0.214–0.383) and inversely correlated with platelet count (r = 0.433, p<0.001, 95% CI -0.019 –-0.010). FIB-4 index showed the highest coefficient correlation level, followed by platelet count. There were no significantly correlations between ALT, HBV DNA levels and WFA^+^-M2BP.

### Correlation between WFA^+^-M2BP level and histopathology

**Fig 1:**
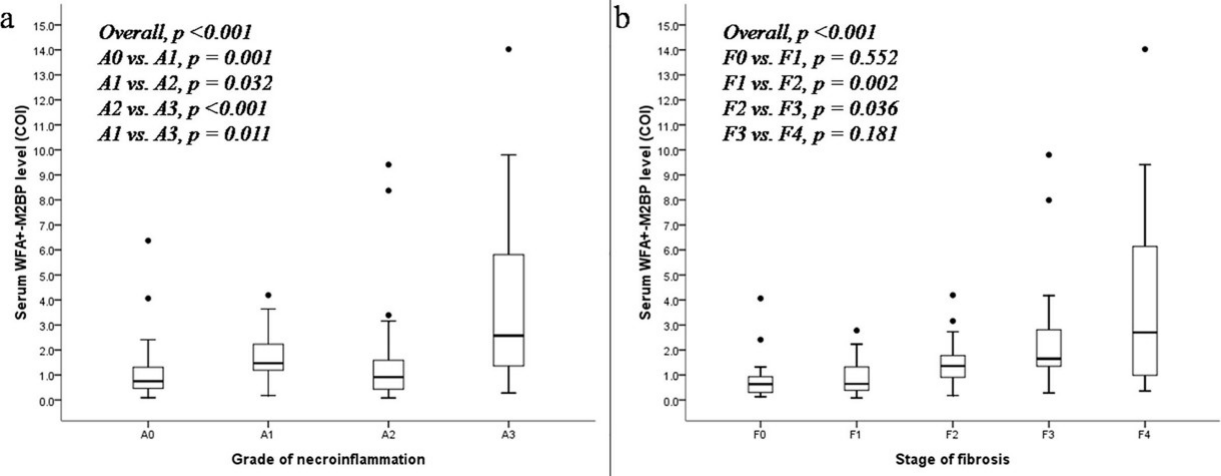
The correlations between serum WFA^+^-M2BP leveland necroinflammation grade (a) and fibrosis stage (b).

The correlations between serum WFA^+^-M2BP leveland necroinflammation grade and fibrosis stage were shown in Fig 1. There was a significant increase in serum WFA^+^-M2BP level according to necroinflammationgrading.(Fig 1A)The median (25^th^- 75^th^ percentile) serum WFA^+^-M2BP levelswere 0.75 COI (0.46–1.32) of A0, 1.47 COI (1.14–2.26) of A1,0.92 COI (0.42–1.63) of A2, and 2.57 COI (1.36–5.88) of A3, respectively(p for linear trend <0.001).

There was a significant correlation between serum WFA^+^-M2BP level and fibrosis stages.(Fig 1B) The median (25^th^- 75^th^ percentile) serum WFA^+^-M2BP levels were 0.63 COI (0.29–1.06) of F0, 0.64 COI (0.38–1.34) of F1, 1.36 COI (0.84–1.80) of F2, 1.65 COI (1.28–3.04) of F3,and 2.70 COI (0.94–6.17) of F4, respectively (p for linear trend<0.001).There was no significant difference of serum WFA^+^-M2BP level according to fibrosis staging when stratified by the clinical profiles such asgender, age, HBeAg, HBV DNA level, and ALT level.

### Comparison of the diagnostic performance of WFA^+^-M2BP, FIB-4, and APRI for liver fibrosis

**Fig 2:**
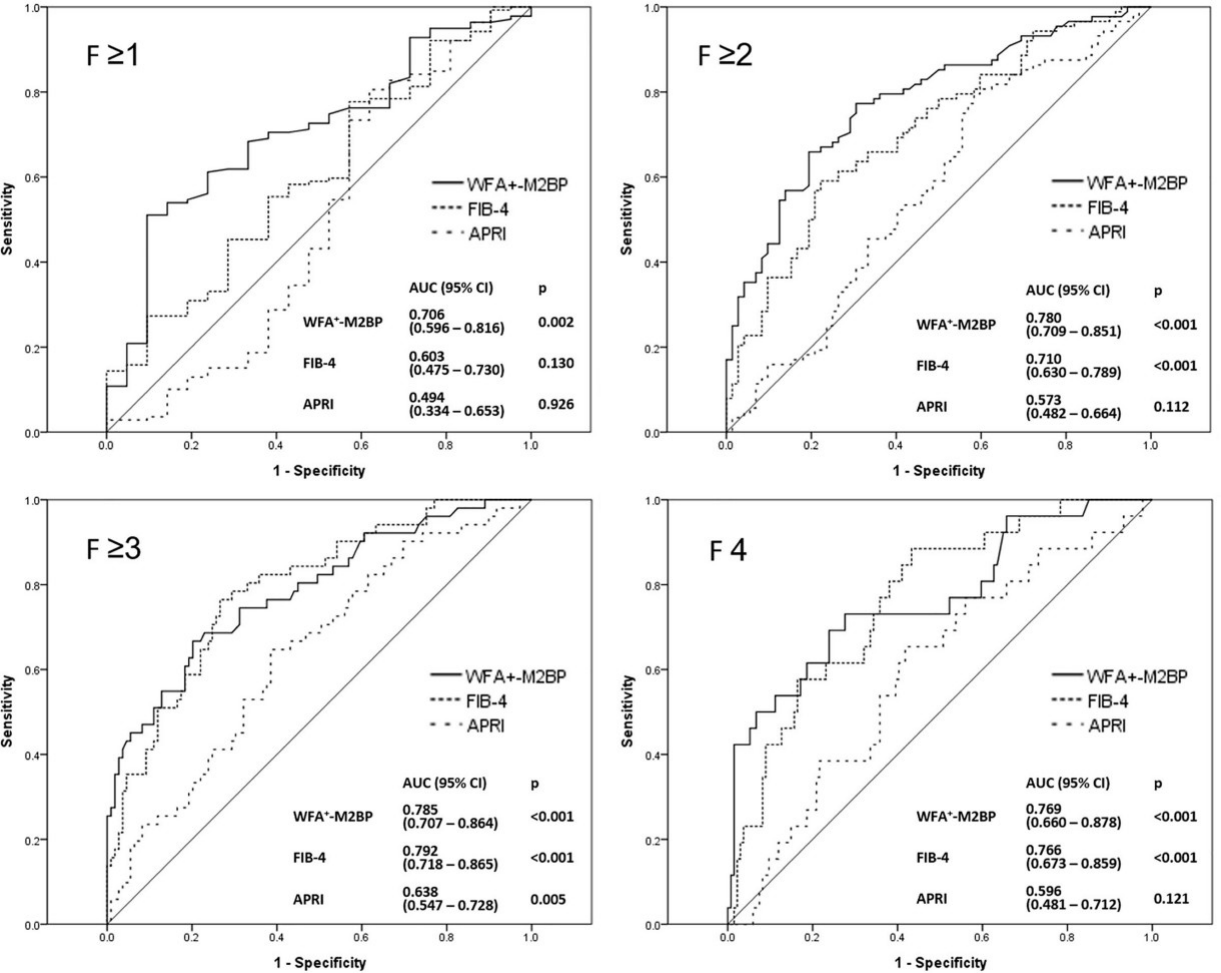
The diagnostic ability of WFA^+^-M2BP, FIB-4, and APRI in the assessment of fibrosis stage.

The diagnostic performance of WFA^+^-M2BP, FIB-4, and APRI in the assessment of fibrosis stage was compared. The accuracyof serum WFA^+^-M2BP level for each fibrosis stage was 0.706 of ≥F1(0.596–0.816, p = 0.002), 0.780 of ≥F2 (0.709–0.851, p <0.001), 0.785 of ≥F3 (0.707–0.864, p <0.001), and 0.769 of F4 (0.660–0.878, p <0.001), respectively. Significant diagnostic accuracy was observed for FIB-4index in assessing fibrosis stages. The significant diagnostic accuracy of APRI index was only observed in assessing fibrosis stage ≥F3 (0.638, 0.547–0.728, p = 0.005). (Fig 2)

Based on Youden’s index from the AUROC, the optimal cutoff value of serum WFA^+^-M2BP level was determined. The optimal cutoff value was for F1 was 0.960, yielding the sensitivity of 61.2%, specificity of 76.2%, positive predictive value (PPV) of 94.4%, negative predictive value (NPV) of 22.9%, and accuracy of 63.1%, respectively. The optimal cutoff value was 1.345for F2, yielding the sensitivity of 65.9%, specificity of 80.6%, PPV of 80.6%, NPV of 65.9%, and accuracy of 72.5%, respectively. The optimal cutoff values for F3 and F4 were 1.535, and 1.665, respectively. The sensitivity, specificity, PPV, and NPV were shown in Table 2.

**Table 2:** Diagnostic performance and optimal cutoff of serum WFA^+^-M2BP level for assessing each fibrosis stage.

	AUC	Cutoff	p	Sensitivity	Specificity	PPV	NPV	Accuracy
F ≥1	0.706	0.960	0.002	61.2%	76.2%	94.4%	22.9%	63.1%
F ≥2	0.780	1.345	<0.001	65.9%	80.6%	80.6%	65.9%	72.5%
F ≥3	0.785	1.535	<0.001	66.7%	79.8%	60.7%	83.7%	75.6%
F 4	0.769	1.665	<0.001	69.2%	76.1%	36.0%	92.7%	75.0%

AUC, area under the receiver operating characteristics curve; PPV, positive predictive value; NPV, negative predictive value.

The factors associated with advanced fibrosis were further analyzed. Inunivariate analysis, age (p <0.001), platelet (p <0.001), ALT (p = 0.015), and WFA^+^-M2BP level (p <0.001) were factors associated with significant fibrosis. The multivariate analysis adjusted above parameters revealed that age (OR 1.05, 95% CI: 1.010–1.092, p = 0.014), platelet count (OR: 0.99, 95%CI: 0.980–0.998, p = 0.013), and WFA^+^-M2BP level (OR: 1.97, 95%CI: 1.299–2.984, p = 0.001) were independently associated factors (Table 3).

**Table 3:** Uni- and multivariate analysis of factors associated with severe liver fibrosis.

	Severe fibrosis (≥ F3)	Non-Severe fibrosis (< F3)		Adjusted		
	N = 51	N = 109	p	OR	95% CI	p
Age, years	52.0 (42.0, 57.0)	37.0 (28.5, 45.5)	<0.001	1.05	1.010–1.092	0.014
Male gender	40 (78.4)	83 (76.1)	0.842			
platelet, x10^3^/μl	137.0 (99.0, 179.0)	207.0 (163.0, 232.0)	<0.001	0.99	0.980–0.998	0.013
AST, U/L	87.0 (52.0, 140.0)	76.0 (54.0, 148.5)	0.895			
ALT, U/L	100.0 (58.0, 224.0)	141.0 (92.5, 245.5)	0.028	1.00	0.997–1.001	0.213
WFA^+^-M2BP, COI	2.11 (1.20, 4.59)	0.86 (0.44, 1.47)	<0.001	1.97	1.299–2.984	0.001
Positive HBeAg	23 (45.1)	33 (30.3)	0.077			
HBV DNA, log_10_ IU/ml	6.3 (4.9, 7.4)	5.7 (3.8, 7.3)	0.152			

AST, aspartate aminotransferase; ALT, alanine aminotransferase; HBeAg, hepatitis B e antigen; WFA^+^-M2BP, wisteria floribunda agglutinin positive Mac-2-binding protein; APRI, aspartate aminotransferase-to-platelet ratio; FIB-4, Fibrosis-4.

### WFA^+^-M2BP combined with age, and platelet to predict advanced liver fibrosis

We further aimed to develop a predictive model for advanced fibrosis. The prior analysis revealed age, platelet, and WFA^+^-M2BP level were the independent factors associated with severe fibrosis. The optimal cutoff value of the independent factors was determined as age of 40 years, platelet of165 x 10^3^/μl, and WFA^+^-M2BP level of 1.535 COI by Youden’s index from the AUROC. The comparison of diagnostic performance of age, platelet, WFA^+^-M2BP level, a combination of the threeparameters, APRI, APRI plus Age, FIB-4, and FIB-4 plus Age score was shown as Table 4. Comparing to each parameter, the combination model showed a better performance in terms of specificity, positive predictive value, and accuracy.

**Table 4:** Diagnostic performance of Age, Platelet, WFA^+^-M2BP, and the combination for assessingadvancedliverfibrosis.

	AUC	p	Sensitivity	Specificity	PPV	NPV	Accuracy
Age >40 years	0.741	<0.001	80.4%	67.9%	53.9%	88.1%	71.9%
Platelet<165x 10^3^/μl	0.739	<0.001	72.5%	75.2%	57.8%	85.4%	74.4%
WFA^+^-M2BP ≥1.535COI	0.785	<0.001	66.7%	79.8%	60.7%	83.7%	75.6%
Combination of Age, Platelet, and WFA^+^-M2BP	0.732	<0.001	51.0%	95.4%	83.9%	80.6%	81.3%
APRI ≥2.0	0.577	0.065	41.2%	74.3%	42.9%	73.0%	63.8%
APRI ≥2.0 plus Age >40 years	0.622	0.013	37.3%	87.2%	57.6%	74.8%	71.3%
FIB-4 ≥3.25	0.691	<0.001	51.0%	87.2%	65.0%	79.2%	75.6%
FIB-4 ≥3.25 plus Age >40 years	0.695	<0.001	49.0%	89.9%	69.4%	79.0%	76.9%

## Discussion

In addition to ultrasound-based methods, several serum biomarkers have been vigorously developing and validating for assessing the degree of fibrosis in the past decade. WFA^+^-M2BP measurement is a newly-developed biomarker which has been validated for disease severity assessment in chronic liver diseases such as chronic hepatitis C and non-alcoholic fatty liver disease. In the present study, we aimed to explorethe performance of serum WFA^+^-M2BP level in the prediction of liver fibrosis in CHB patients. Our results demonstrated that serum WFA^+^-M2BP is competent in evaluating disease severity in CHB. We also demonstrated a model of combined age, platelet, and WFA^+^-M2BP to accurately predict advancedliver fibrosis. Our results thus provided the evidence for clinical application of combined non-invasive tools in this aspect.

The main pathogenic mechanism of hepatocarcinogenesis is underlying necroinflammation and its subsequent fibrogenesis. Therefore, severity estimation ofliver fibrosis is important for treatment efficacy, prognosis, HCC surveillance and determining the optimal treatment strategies. Liver core biopsy is much hurdled by the potential complications, intra-observer variability, sampling errorsand patients’ willingness.[25–27]The investigation of non-invasive methods is a feasible way in a clinical setting. WFA^+^-M2BP is a newly developed serum biomarker for liver fibrosis assessment. M2BP is a glycoprotein which secreted from many cells, but mainly from hepatocytes. Fluctuations of M2BP in the sera increase following the progression of liver fibrosis. The diagnostic accuracy and clinical manifestation of WFA^+^-M2BP had been well reported in patients with chronic hepatitis C, non-alcoholic fatty liver disease, and in the prediction of HCC development or recurrence.[18,20,21,28–31]

Liver fibrosis stage should be taken into consideration regarding the initiation of antiviral therapy among CHB patients according to current regional guidelines. The performance of WFA^+^-M2BP in the diagnosis of liver fibrosis in CHB patients had been reported in previous studies. In most of the studies, WFA^+^-M2BP was reported to have similar diagnostic efficacy with other biomarkers in distinguishing each fibrosis stage.[32–34]Our results echoed the previous study conducted by Zou et al showing that WFA^+^-M2BP asa useful marker to assess liver fibrosis in CHB patients.[35] We also developed a model combined WFA^+^-M2BP, age, and platelet to better predict advanced fibrosis which was not reported in the previous studies. Although the sensitivity of the combination model was only 51.0%, the combination model still could distinguish patients who werereally not advanced fibrosis because of the high specificity of the combination model. The result also showed a better diagnostic performance than APRI, FIB-4, or their combination index. To our knowledge, the present study was the first study addressing the combination of WFA^+^-M2BP and other markers to accurately predict liver fibrosis. In the future, a prospective, larger scale of study is needed for further validation of the novel findings of the current study.

The elastography, such as Fibroscan, which measured the liver fibrosis directly had been well developed. It would be a more direct and meaningful comparison to comparing WFA^+^-M2BP against Fibroscan. However, we did not perform the comparison because of the unavailable equipment at the time of data collection. To differentiate advanced fibrosis, FIB-4 ≥3.25 and APRI ≥2 were well studied and accepted values. We compared the diagnostic performance of the combination model with the established FIB-4, APRI, and with age combination model. The results showed a better performance using the WFA^+^-M2BP combination model. The WFA^+^-M2BP itself had shown a better predictive value than FIB-4 and APRI. That might explain why the WFA^+^-M2BP combination model was better than FIB-4 or APRI combination model. Although that, a further large scale study remains needed to confirm the results.

In conclusion, serum WFA^+^-M2BP level was a useful non-invasive marker for liver fibrosis assessment. The combination of age, platelet, and WFA^+^-M2BPprovided a better model to determine advanced liver fibrosis in CHB patients.

## Supporting information

S1 File Dataset of all enrolled patients. (XLSX)
